# Unexpected and striking effect of heparin-free dialysis on cytokine release

**DOI:** 10.1007/s11255-017-1589-8

**Published:** 2017-04-19

**Authors:** Alicja Rydzewska-Rosolowska, Joanna Gozdzikiewicz-Lapinska, Jacek Borawski, Ewa Koc-Zorawska, Michal Mysliwiec, Beata Naumnik

**Affiliations:** 1 I Department of Nephrology and Transplantation With Dialysis Unit, Medical University of Bialystok, Zurawia 14, 15-540, Białystok, Poland; 2 II Department of Nephrology and Hypertension With Dialysis Unit, Medical University of Bialystok, Białystok, Poland

**Keywords:** Monocyte chemoattractant protein-1, Endostatin, Activin A, Enoxaparin, Heparin-grafted membrane, Hemodialysis

## Abstract

Heparin (both unfractionated and low molecular weight) is not only a potent anticoagulant but also has many pleiotropic effects, some of which are mediated by cytokine release. We compared the effect of hemodialysis (HD) with enoxaparin as an anticoagulant and without systemic anticoagulation (heparin-grafted membrane—Evodial) on the release of monocyte chemoattractant protein 1 (MCP-1), endostatin (ES) and activin A (Act-A). Nineteen stable HD patients were dialyzed with or without heparin, and plasma levels of MCP-1, ES and Act-A were measured after such a dialysis. During HD with Evodial, the intradialytic levels of all three cytokines were 2–3 folds lower. The between-anticoagulant differences were significant over time for all three cytokines: MCP-1 (*P* < 0.001), ES (*P* < 0.001) and Act-A (*P* < 0.001). This striking effect of heparin-free dialysis with Evodial membrane may be beneficial not only because it reduces the possibility of bleeding complications but also because it might reduce proinflammatory cytokine concentration and therefore contribute to the improvement in endothelial function. Further studies are needed to determine whether it has a positive effect on morbidity and mortality of maintenance HD patients.

## Introduction

Heparin, both unfractionated (UFH) and low molecular weight (LMWH) is not only a potent anticoagulant but also has many pleiotropic effects, some of which are mediated by cytokine release [1–3]. Hemodialysis (HD), a procedure during which heparin is routinely used, can also cause an increase in cytokine levels. Therefore, it generates an abundance of growth factors, cytokines and other mediators playing an important role in the pathogenesis of many signs, symptoms and complications of end-stage renal disease. To finally elucidate between the role of heparin and hemodialysis on cytokine profiles, aiming to prove that heparin-free dialysis does not cause cytokine release, we constructed the current study to directly compare the effect of dialysis using Evodial membranes with standard dialysis with LMWH anticoagulation. Based on our former experience, we chose to measure monocyte chemoattractant protein-1 (MCP-1), endostatin (ES) and activin A (Act-A) concentrations [4, 5].

Microinflammation is practically universally present in all hemodialysis patients. It results from serious disturbances of acquired immunity which are caused by many different factors. The main players are: uremia per se, medications used and the hemodialysis procedure and its complications. Antigen-presenting cells (monocytes) pre-activation, overproduction of proinflammatory cytokines (Il-1, Il-6, TNF-α) and deficient T lymphocyte-dependent immune response are causes of frequent infections, malnutrition-inflammation-atherosclerosis (MIA) syndrome, high failure vaccination rates among others [6].

The effect of low molecular weight heparins on the above-mentioned microinflammation is not fully understood. Most groups (including ours) report beneficial effects of different kinds of heparins in HD patients. However, there are some studies in other patients showing other results, e.g., an Australian group demonstrated that enoxaparin decreased by 48% and dalteparin increased by 25% the release of proinflammatory cytokines from peripheral blood mononuclear cells of asthmatics [7]. Therefore, we aimed to further elucidate the subject.

Monocyte chemoattractant protein-1 belongs to the family of chemokines—small heparin-binding proteins that regulate cell trafficking. It is produced, among others, by epithelial, endothelial, mesangial, smooth muscle cells and fibroblasts and controls the migration and infiltration of monocytes, memory T lymphocytes and natural killer cells. Evidence points to its major role in many human pathologies include atherosclerosis (causes plaque instability), rheumatoid arthritis and progressive renal injury in diabetic nephropathy [8]. The effect of heparin on MCP-1 is unknown as the results are conflicting [9–11].

Endostatin is a potent angiogenesis inhibitor. It is a fragment of collagen XVIIIA1—a proteoglycan containing heparan sulfate. It has a number of biological functions: It suppresses tumor cell migration and invasion, inhibits endothelial cell migration and proliferation, acts against vascular endothelial growth factor, and induces endothelial cell apoptosis and many others [12]. ES was named “resistance-free cancer therapy” and has been extensively demonstrated to inhibit tumor growth in many models with absolute lack of toxicity [13]. Its benefits were also demonstrated in other diseases, e.g., diabetic nephropathy [14]. Endostatin levels are remarkably high in HD patients and apparently not related to heparin administration [15], and in this, population may unfortunately be a marker of “defective” angiogenesis causing a higher rate of genitourinary malignancies (its levels are elevated in renal cell carcinoma and correlate with tumor aggressiveness) [16].

Activin A was discovered in the 80s as a gonadal protein. It is a pluripotent cytokine from the transforming growth factor-β family, which takes part in organogenesis, wound healing, fibrosis, immune response, pancreatic β-cell number and function regulation. It is also proven that heparin releases Act-A during hemodialysis [4, 5].

## Patients and methods

### Patients

Nineteen stable, chronic hemodialysis patients participated in the study. The main exclusion criteria were: lack of consent, history of neoplasm, leukopenia, ongoing infection, trauma or surgery (up to one month before study enrollment), immunosuppressive treatment, hemodialysis vintage shorter than 3 months, lack of native arteriovenous fistula, *Kt*/*V* < 1,2, hemostatic abnormalities and oral anticoagulation. The basic clinical data are presented in Table 1.

**Table 1:** Clinical characteristics of the patients

Age (years)	57 (18–81)
*Sex*	
Male (*n*/%)	9/47.4%
Female (*n*/%)	10/52.6%
*Cause or renal disease*	
Unknown (*n*/%)	8/42.1%
Glomerulonephritis (*n*/%)	4/21%
Polycystic kidney disease (*n*/%)	3/15.7%
Diabetic nephropathy (*n*/%)	1/5.3%
Chronic interstitial nephritis (*n*/%)	1/5.3%
Hypertensive nephropathy (*n*/%)	1/5.3%
Obstructive nephropathy (*n*/%)	1/5.3%
Dialysis vintage (months)	42 (7–119)
*Major comorbidities*	
Hypertension	17/89.4%
Cardiovascular disease	6/31.5%
Diabetes	1/5.3%
*Type of dialysis membrane*	
Polysulfone	16/84.2%
Modified cellulose	3/15.8%

The study was conducted according to Good Clinical Practice and in agreement with Declaration of Helsinki. The protocol was approved by local bioethics committee, and all individual participants signed informed consent before entering the study. The study was a prospective one planned initially to study the effect of heparin-free dialysis on myeloperoxidase release [17]; for this study, frozen samples were used (19 for MCP-1 and endostatin and 12 for activin A).

### Study design and sampling

All participating patients were dialyzed with a stable, clinically efficient dose of enoxaparin (Clexane, Sanofi-Aventis, France), administered as a single bolus, before commencing the study (doses ranged between 20 and 60 mg). During the study, dialysis dose, dialyzer type (reported in Table 1) and other pharmacologic treatment remained unchanged. At least 48 h after a dialysis session during which enoxaparin was used, the patient was switched to dialysis without systemic anticoagulation. During this procedure, a polycarbonate hollow-fiber dialyzer with heparin-grafted membrane (Evodial, Gambro-Diaverum, Lund, Sweden) was used (the area of the dialyzer was kept constant). Before both procedures, the extracorporeal circuit was flushed with 1000 ml of normal saline containing 2.0 IU/ml of unfractionated heparin to avoid early clotting. Blood was drawn from the arterial line before the start of dialysis procedure and, respectively, after 10 (T10) and 120 min (T120) after slowing the blood flow to 100 ml/min for 1 min.

### Laboratory parameters

Plasma levels of MCP-1, ES and Act-A were measured in duplicate, by ELISA, using commercially available kits from R&D Systems and Oxford Bio-Innovation Ltd., respectively, according to manufacturer’s instructions. For calculation of results, a computer and curve-fitting program were used. The within- and between-assay coefficients were <8%.

### Statistical analysis

Data were analyzed using Stata software (version 9.2). The data distribution was normal, and one-way or two-way ANOVA were used as appropriate. For post hoc group comparisons, Bonferroni correction was used. All statistical tests were two-sided, and *P* < 0.05 was considered significant.

## Results

**Fig. 1:**
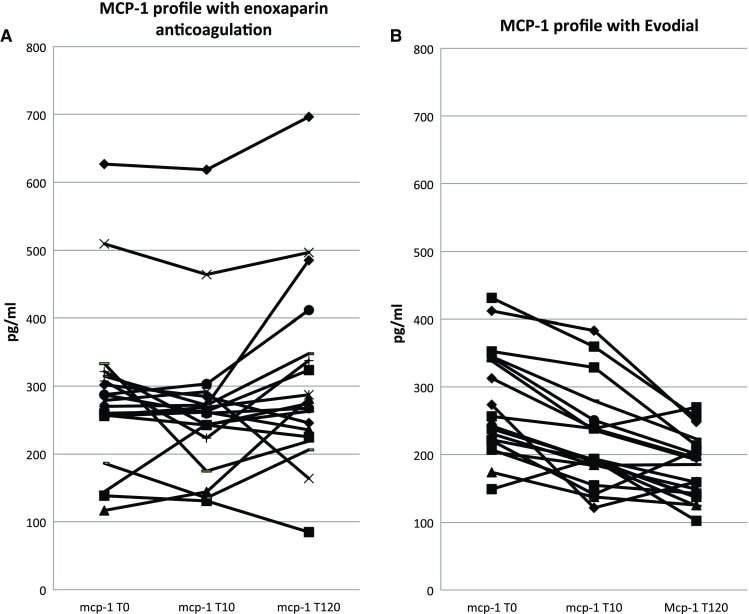
MCP-1 profiles during enoxaparin and Evodial dialysis

During standard dialysis with enoxaparin plasma, MCP-1 levels remained stable (*χ* ^2^ = 0.792, *P* = 0.613, Fig. 1a). Pre-dialysis, they were 287.1 pg/ml (sd 119.1), at T10 268.1 pg/ml (sd 112.5) and at T120 307.9 pg/ml (sd 137.9).

**Fig. 2:**
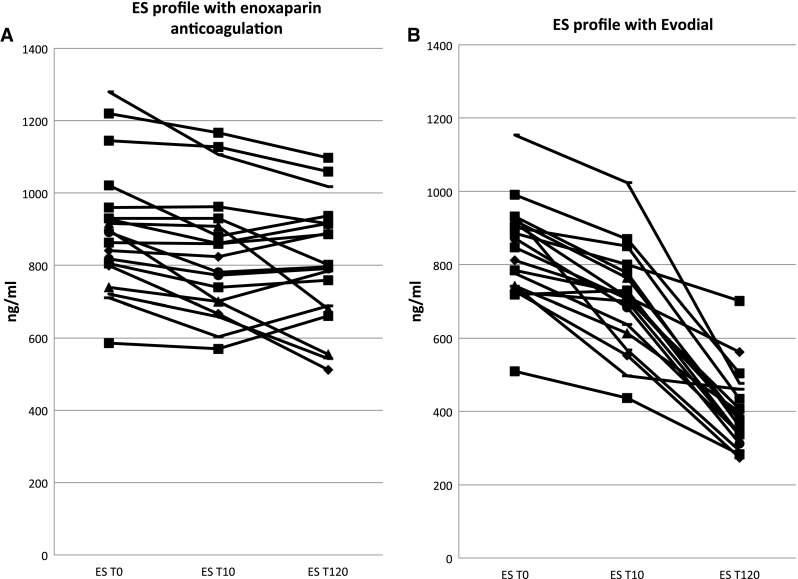
ES profiles during enoxaparin and Evodial dialysis

Plasma levels of ES also remained constant during the entire procedure (*χ* ^2^ = 0.013, *P* = 0.239, Fig. 2a). They were 898.1 ng/ml (sd 174.8) at T0, 832.3 ng/ml (sd 172.9) at T10 and 804.6 (sd 170.1) at T120.

**Fig. 3:**
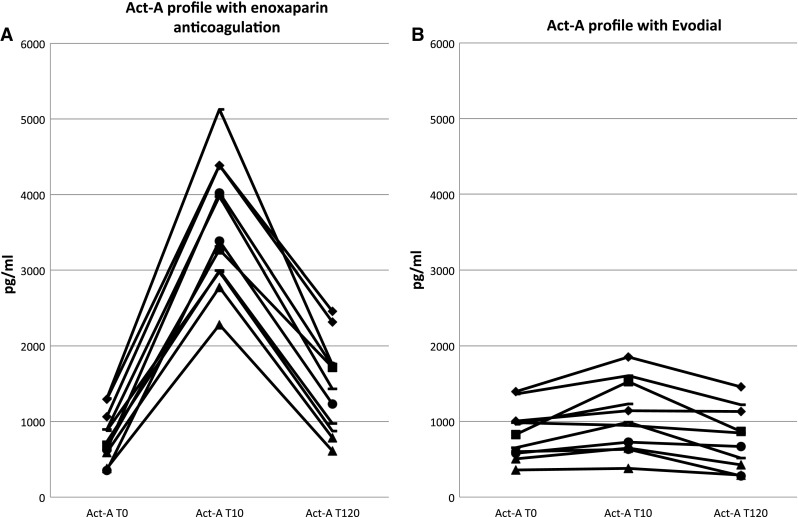
Act-A profiles during enoxaparin and Evodial dialysis

Act-A levels changed remarkably over time (*χ* ^2^ = 7.724, *P* < 0.001, Fig. 3a). Before the start of hemodialysis, the mean level was 797.7 pg/ml (sd 325.9), and then, it increased abruptly (in all patients) to a mean of 3596.8 pg/ml (sd 849.1) (*P* < 0.001). After 120 min, it remained elevated (*P* = 0.07) with a mean level of 1443.3 pg/ml (sd 613.2).

The most striking changes were seen with Evodial dialysis. Pre-dialysis values of all three cytokines were comparable. MCP-1 decreased over time (*χ* ^2^ = 4.394, *P* < 0.001, Fig. 1b) from 273.5 pg/ml (sd 78.7) to 220.9 pg/ml (sd 73.3) at T10 (*P* = 0.063) and 180.4 pg/ml (sd 48.2) at T120 (*P* < 0.001).

ES decreased from 835.9 ng/ml (sd 136.1) at T0 to 703.0 (sd 137.4) at T10 (*P* = 0.007) and 400.5 (sd 105.9) at T120 (*P* < 0.001). The changes were statistically significant (*χ* ^2^ = 1.435, *P* < 0.001, Fig. 2b).

Act-A levels, contrary to enoxaparin dialysis, remained constant over time (*χ* ^2^ = 0.889, *P* = 0.241, Fig. 3b) at 838.9 pg/ml (sd 338.9) pre-dialysis, 1060.5 pg/ml (sd 460.8) after 10 min (*P* = 0.629) and 770.5 pg/ml (sd 405.9) after 120 min (*P* = 1.00).

The between-anticoagulant differences were significant over time for all three cytokines: MCP-1 (*P* < 0.001), ES (*P* < 0.001) and Act-A (*P* < 0.001).

## Discussion

Our study reports unexpected depletion of plasma levels of MCP-1, endostatin and activin A during heparin-free systemic hemodialysis when compared to standard dialysis with low molecular weight heparin (in this case enoxaparin). The effect of Evodial membranes on ES and Act-A was to our knowledge not reported before, and the effect on MCP-1 is consistent with data published by Morena et al. [18].

MCP-1 levels during dialysis remained stable in our study, although there was a tendency to their intradialytic lowering (after 10 min) with a subsequent rise at 120 min. This is in agreement with our previous study [11]; most papers report a stable or slightly increased levels [18–20] attributing the effect to dialysis itself (inadequate clearance or enhanced synthesis and release) rather than heparin used. It is widely believed that MCP-1 is a marker of biocompatibility and that its increased levels contribute to inflammation, dyslipidemia and atherosclerosis—the main burden of end-stage renal disease patients. The potential of Evodial for lowering circulating MCP-1 levels could have beneficial effects on these conditions. The probable mechanism is adsorption of MCP-1 onto the heparin-grafted membranes. In our study, during Evodial dialysis, heparin was used only for pre-rinsing (in contrast to the study by Morena and co-workers who used either a 100 or 30% dose of heparin during Evodial dialysis), and therefore, it could not contribute to the release of MCP-1.

ES levels were, as reported before [15], extremely high in our patients and did not change after heparin administration. As was the case with MCP-1 levels, they decreased dramatically during Evodial dialysis. The phenomenon of very high endostatin levels in kidney impairment is intriguing. On one side, it could suggest an anti-tumor activity, and on the other, a much probable theory is that it reflects defective angiogenesis, may be a cause for defective collateral vessel formation and contribute to the higher rate of genitourinary tract malignancies observed in HD patients [21]. ES levels are also increased in patients with renal cell carcinoma [16]. Therefore, the potential benefit of lowering endostatin levels during hemodialysis could be of paramount importance for these patients.

The effect of heparins (both UFH and LMWH) on Act-A has been studied extensively by our group and others [4, 5]. The mechanisms mediating this phenomenon are probably displacement from cell surface proteoglycans [22] and dissolution of the complex [23]. As bioactive activin A is detected in atherosclerotic plaques, it causes smooth muscle cell proliferation and differentiation of monocytes into macrophages and probably promotes the development of atherosclerosis [24]. It also plays an important role in modulating the immune system controlling the release of other proinflammatory cytokines; therefore, blocking its actions may be of therapeutic interest in inflammatory conditions [25]. Heparin-free dialysis prevented activin A release mediated by enoxaparin and stabilized its levels.

Other cytokines studied during Evodial dialysis were interleukin 6 (Il-6), tumor necrosis factor-α (TNF-α) [18] and myeloperoxidase (MPO) [17]. Il-6 and TNF-α levels remained stable during dialysis, but MPO levels were significantly lower. This suggests that heparin-grafted membrane has a potential of binding other cytokines as well and it is not a solitary phenomenon. Further studies are needed to elucidate the role of this membrane, as it is also possible that it will bind some anti-inflammatory cytokines.

We are of course aware of the limitations of our study, mainly the small number of patients, although since they served as their own controls it could be minimized. The other limitation would be that only one dialysis with Evodial was performed, but since the effect was abrupt and already seen after 10 min of procedure we believe there was no carry-over effect or it was minimal. It is also unknown what happens to cytokine levels after the end of dialysis treatment, but because this procedure is carried out three times weekly one can speculate that cytokine depletion is of clinical importance. Last but not least, we did not study other proinflammatory cytokines, and obviously, decreased levels of those cytokines do not necessarily imply “less” inflammation, but the notion is intriguing and definitely requires more research.

In conclusion, heparin-free dialysis with Evodial membrane may be beneficial not only because it reduces the possibility of bleeding complications, but also because it might reduce the level of proinflammatory cytokines and therefore contribute to the improvement in endothelial function. Further studies are needed to determine whether it has a positive effect on morbidity and mortality of maintenance hemodialysis patients.
